# Supplementary material for: The association between urbanization and adolescent depression in China
Source: PeerJ. 2024 Feb 22;12:e16888. doi: 10.7717/peerj.16888 (PMC10894590; doi:10.7717/peerj.16888)
Supplement: Supplemental Information 3 [file peerj-12-16888-s003.docx]

**Depression:**

No (0)

Yes (1)

**Gender:**

Female (0)

Male (1)

**Ethnicity code:**

Minorities (0)

Han (1)

**Level of education:**

Primary school or less (0)

Junior high (1)

High school (2)

College or more (3)

**Marital status:**

Single (0)

Married (1)

**Urban/rural areas:**

Rural areas (0)

Urban areas (1)

**BMI:**

<18.5 (0)

18.5-24.0 (1)

24.0-28.0 (2)

≥28.0 (3)

**Self-rated health:**

poor health (0)

fair (1)

good (2)

very good (3)

excellent (4)

**Smoking:**

No (0)

Yes (1)

**Drinking:**

No (0)

Yes (1)

**Exercise:**

No (0)

Yes (1)
